# Patient Preferences for Lifestyle Management in a Multi-site Randomized Lifestyle Trial for Remission of the Metabolic Syndrome

**DOI:** 10.1007/s12529-024-10278-2

**Published:** 2024-03-22

**Authors:** Katherine Iannuzzelli, Sumihiro Suzuki, Kelly Karavolos, Lynda H. Powell

**Affiliations:** Department of Preventative Medicine, Rush University Medical Center, Chicago, IL USA

**Keywords:** Patient preference, Randomized behavioral controlled trial, Metabolic syndrome, Lifestyle treatment

## Abstract

**Background:**

Randomized behavioral clinical trials are the gold standard for evaluating efficacy of a behavioral treatment. However, because participants are generally unblinded to treatment, preference for a specific treatment option can lead to biased results and/or reduced treatment efficacy. The purpose was to describe the relative frequency and correlates of existence of a preference and patient preference for either an in-person group-based or a remote self-directed, lifestyle treatment prior to randomization to one of these treatments.

**Methods:**

The Enhanced Lifestyles for Metabolic Syndrome (ELM) trial is a multi-site behavioral clinical trial that compares efficacy of a group-based vs. a self-directed approach to lifestyle change on 2-year remission of the metabolic syndrome. Prior to randomization, participants were asked whether they had a preference for a particular treatment and, if so, which approach they preferred. Baseline data were used for a series of logistic regression models to determine behavioral correlates of treatment preference, independent of socioeconomic factors.

**Results:**

Of the 331 participants, 131 (39.6%) had no preference for either treatment. Among the 200 with a preference, 56 (28.0%) preferred the self-directed program. Strength of a pre-existing habit of eating vegetables on most days was an independent correlate of no preference (adjusted OR, 1.27; 95% CI, 1.01–1.61; *p* = 0.03) and preference for a self-directed program (adjusted OR, 1.55; 95% CI, 1.09–2.22; *p =* 0.01).

**Conclusion:**

A pre-existing habit of eating vegetables was associated with no preference and preference for a less intensive lifestyle treatment. Post-treatment follow-up of the trial results will determine if concordance between preference and treatment assignment influences outcomes.

## Introduction

Randomized controlled trials (RCTs) are the gold standard design for studying a causal relationship because they minimize confounding, avoid selection bias, and provide the strongest evidence for a cause-effect relationship between a treatment and outcome [1]. When the RCT tests a behavioral treatment, difficulties in blinding to treatment assignment pose a unique challenge. If participants have a preference for a particular treatment, external validity could be threatened if they refuse to be randomized because of the risk that they might not receive their preferred treatment. Internal validity could be threatened if participants with a preference agree to be randomized, receive their non-preferred treatment, and drop out, are non-adherent, crossover to get the preferred treatment on their own, and/or fail to complete follow-up exams [2, 3]. Given these potential threats to the validity of a behavioral clinical trial, it is important to understand the nature of patient preference and how it might influence the estimate of therapeutic benefit [4]. 

Preference for a particular treatment, studied in a systematic review of 11 fully randomized, single-blind RCTs that included participants’ baseline preference for treatment group, showed that 56% of participants had a preference [2]. Given this percentage, the main question of interest is whether people who are generally willing to express a preference differ from people who are typically indifferent [4].

This question concerns the characteristics of those who do and do not have a preference. Having no preference for a particular treatment has been found to be more common in males and those with more education [3, 5]. There are few data on the impact of factors such as support from family or friends on preference for treatment. In a consensus study that drew on systematic reviews and input from experts, social support was relegated to the category of “inconclusive” with no data available [6].

The purpose of this paper was to investigate the question of prevalence and predictors of patient preference in a large, multi-site lifestyle trial of patients with the MetS. The ELM (*E*nhanced *L*ifestyles for the *M*etabolic Syndrome) trial aims to compare two lifestyle treatments—an in-person group-based program vs. a remotely delivered self-directed program—on sustained 2-year remission of the MetS. Patient preference for these two treatment programs was assessed before randomization but after participation in an information session where the details of what was required in each of the two treatments were presented. The first aim was to determine the relative frequency of a preference for either of these two treatments vs. no preference and the correlates of that preference. The second aim focused on the subgroup of participants with a preference and examined the relative frequency and correlates of a specific preference for one of the two available arms.

The current study is the first step of an exploration of the relative frequency, correlates, and impact of patient preference in a behavioral trial on clinical outcomes over 24 months. This work could inform behavioral trial design by determining whether preference should be considered in tailoring the intensity of treatment, the randomization strategy, and pre-planned subgroups. It can inform clinical practice by helping healthcare providers better align treatment to patients, consistent with precision medicine approaches [7].

## Methodology

### Study Population

The sample included 331 individuals from the five sites in the ELM multi-site trial: Rush University Medical Center in Chicago IL, Rochester Regional Health in Rochester NY, University of Colorado Anschutz Medical Campus in Denver CO, University of Missouri-Kansas City MO, and Geisinger Health System in central and eastern PA. Eligible participants were men and women, 18 years or older, diagnosed with the MetS defined by the Joint Interim Statement of the International Diabetes Federation Task Force on Epidemiology and Prevention, National Heart, Lung, and Blood Institute, American Heart Association, World Heart Federation, International Atherosclerosis Society, and International Association for the Study of Obesity [8]. Additional eligibility included participants who are interested in lifestyle change to manage the MetS and do not have any medical contraindications or logistical barriers. At the time of the current study, recruitment of the target population of 600 was ongoing, but recruitment procedures were constant throughout the trial and participants yet to be recruited would be unlikely to differ from those in the sample presented. Recruitment occurred in waves of approximately 150 patients/wave, with each of five sites recruiting 30 participants/wave.

Once a site recruited 30 eligible participants, they were randomized in a 1:1 ratio to the group-based or self-directed program. After the initial screen for eligibility but prior to randomization, potential participants are asked to attend a group-based information session where the details of each treatment program were explained, and then to respond to a question about their preference for treatment (group-based, self-directed, or no preference).

### Measures

Screening and enrollment into the trial was conducted in three steps: initial screen, information session, and baseline exam. All variables were assessed during the baseline exam and before randomization. The evaluated measures were chosen based upon initial hypotheses that can be broadly categorized into the following: (1) participants with greater familial commitments and perceived stress would prefer self-directed treatment due to competing responsibilities; (2) participants with automatic healthy habits, greater support for those habits, and greater quality of life would prefer self-directed treatment due to perception of not needing additional support from professionals or a group. In addition, we included all important sociodemographic variables.

#### Patient Preference for Treatment Arm

To maximize true informed consent, all prospective patients underwent a group-based information session where the requirements for participation in the trial were described, details about the two trial programs were provided, and participants were encouraged to reflect on their personal pros and cons of participation [9]. If prospective participants remained interested in participating in the trial, they were scheduled for a baseline visit. During this visit, they were asked the following: Even though you will be randomly assigned to only one of these two programs (based purely on chance), we want to know whether you have a preference for one program or another. Which of the following statements do you agree with: a) I prefer to be in the group-based program, b) I prefer to be in the self-directed program, c) I don’t have a preference between the two groups. 

##### Quality of Life

The SF-36 Version 1 Survey [10] was used to assess two subscales: Vitality and Mental Health. Both subscales ranged from 0 to 100 with higher scores reflecting healthier status.

##### Healthy Habits

The Self-Report Habit Index [11] was used to assess the strength of a daily habit in four areas that were tailored to the goals of the trial: eating a half-plate of vegetables, ≥ 30-min brisk walk, pause before reacting emotionally, and sensory awareness during cooking, eating, and walking. Strength was defined as whether or not the habit was an automatic part of the daily routine and measured for each of the four goals by five questions (e.g., “It is part of my daily routine,” “I do it without thinking about it.”). Each question used a 5-point Likert scale to express automaticity. The answers to the five questions were averaged within each of the four goals to obtain a habit strength score ranging from 1 to 5 where higher scores indicated stronger habit strength.

##### Social Support for Diet and Physical Activity

The Social Support for Diet and Exercise Survey [12] was used to measure perceived social support from family and friends for health-related eating and exercise behaviors. Five validated subscales were used in this analysis: family encouragement for healthy diet (range 5–25), friend encouragement for healthy diet (range 5–25), friend participation in exercise (range 10–50), family participation in exercise (range 10–50), and family rewards for exercise (range 3–15). Higher scores indicate higher social support.

##### Perceived Stress

The Perceived Stress Scale [13, 14] is a 14-item scale assessing the degree to which situations in a person’s life in the past month were perceived as stressful (e.g., upset because of something that happened unexpectedly; unable to control important things in your life). A total score was created using the mean of 14 items, each of which was scored on a 5-point Likert scale. Scores ranged from 0 to 56, with higher scores corresponding to greater perceived stress.

##### Number of Adults Living in Home

This variable measured adults living in the home aged 18 years or older and included spouse/partners, parents, grandparents, sons/daughters (including in-law, adopted, step or foster), brothers/sisters (including in-law, adopted, step or foster), grandchildren, and other relatives.

##### Number of Children Living in Home

This variable included children under 18 living in the home in the form of sons/daughters (including in-law, adopted, step or foster), brothers/sisters (including in-law, adopted, step or foster), grandchildren, and other relatives. One participant reported an exchange student living in the home and was included in this category.

##### Date of Baseline Survey

This was the date when participant preference was reported. To examine the impact of the evolving pandemic on preferences, these dates were combined into three categories. (1) Pre-COVID: before March 11, 2020, when the World Health Organization (WHO) declared coronavirus disease as a pandemic. (2) During Peak: between March 12, 2020, and May 1, 2021. (3) After Peak: after May 1, 2021, when the Centers for Disease Control and Prevention (CDC) changed its guidance so that fully vaccinated individuals need not wear masks in most situations.

#### Sociodemographic Variables

##### Age and Years of Education

Were modeled continuously.

##### Gender

Was self-reported as female, male, non-binary, or refused. For analyses, gender was collapsed into two categories of female and male, resulting in the exclusion of two participants.

##### Race/Ethnicity

Was a combined measure. Race was measured from an eight-category question (Black/African American, American Indian/Alaskan Native, Asian, Native Hawaiian/Pacific Islander, Caucasian/White, Multi-Ethnic/Mixed Race, Other, and Refused). Ethnicity was measured from a two-category question (Hispanic/Latino/Spanish origin (Yes, No)). Responses were categorized into four race/ethnicity categories: Hispanic, Black/African American (non-Hispanic), Caucasian (non-Hispanic), and Other (non-Hispanic). Three participants refused to answer one or both questions. For the multivariable binary logistic regression models, we collapsed into two categories: Caucasian (non-Hispanic) and Non-Caucasian.

##### Ability to Pay for Basics

This was reported in five categories: Very hard, Somewhat hard, Not hard at all, Refused, and Don’t know. For analyses, the data were collapsed into two categories: Very hard/Somewhat hard and Not hard at all. Two participants did not respond to this question and were excluded.

##### Relationship Status

Was reported in seven categories: Single, Living with partner, Living with spouse, Living separately from spouse, Divorced, Widowed, and Refused. For analyses, the data were collapsed into three categories: Single, Living with spouse/partner, Divorced/Widowed/Living separately from spouse.

### Analysis

All analyses were conducted using SPSS version 26. Analyses of preference vs. no preference were conducted on the full sample of 331. Analyses of preference for group-based vs. self-directed were conducted on the subsample of 200 participants who had a preference. Descriptive statistics were calculated for the total cohort and the cohort stratified by treatment preference. Unadjusted comparisons were evaluated using t-tests for continuous variables and chi-squared or Fisher’s exact tests for categorical variables. Adjusted associations were evaluated with multivariable binary logistic regression models using patient preference as the outcomes for all variables that were found to affect preference in the unadjusted comparisons. All models included the sociodemographic covariates of gender, age, ability to pay for basics, education, race/ethnicity, and relationship status. Serial modeling was used. The base model included the sociodemographic correlates alone. Subsequent models added each hypothesized bivariate correlate to the base model. The final model included all the bivariate correlates to the base model to assess their independence from one another. All associations that were significant at the 0.05 level of significance were noted in table.

## Results

### Description of the Population

There were 331 participants, of which 200 (60.4%) had a preference for their treatment. Of the 200 participants with a preference, 144 (72%) preferred a group-based program and 56 (28%) preferred a self-directed program. The average age was 55 years, 241 (72.8%) identified as female, and 212 (64.6%) identified as Caucasian, non-Hispanic. The average years of education was 15.9, and a majority (85.2%) had no difficulty paying for basic needs.

### Preference vs. No Preference

Table 1 shows descriptive statistics for the total cohort and unadjusted comparisons between participants with and without a preference for a particular treatment. Those with no preference had fewer years of education (15.6 vs. 16.2, *p* = 0.04), a stronger habit of eating vegetables on most days (3.1 vs. 2.8, *p* = 0.02), and more encouragement for eating a healthy diet from friends (11.4 vs. 10.3, *p* = 0.04) compared to those with a preference.

**Table 1:** Baseline characteristics in total cohort and comparing participants with and without a preference for a particular treatment

	**Total** ***N* = 331**	**Preference** ***N* = 200**	**No preference** ***N* = 131**
Age (years), mean (sd)	55.3 (11.0)	55. 9 (11.2)	54.4 (10.6)
Female, *N* (%)	241 (72.8%)	142 (71.7%)	99 (75.6%)
Education (years), mean (sd)	15.9 (2.8)	16.2 (2.8)*	15.6 (2.7)*
Race/ethnicity, *N* (%)			
Hispanic	32 (9.8%)	15 (7.6%)	17 (13.1%)
Black or African American, non-Hispanic	68 (20.7%)	44 (22.2%)	24 (18.5%)
Caucasian, non-Hispanic	212 (64.6%)	126 (63.6%)	86 (66.2%)
Other, non-Hispanic	16 (4.9%)	13 (6.6%)	3 (2.3%)
Refused	3 (0.9%)	2 (1%)	1 (0.8%)
Ability to pay for basics, *N* (%)			
Very hard/somewhat hard	47 (14.2%)	29 (14.5%)	18 (14.0%)
Not hard at all	282 (85.2%)	171 (85.5%)	111 (86.0%)
Refused	2 (0.6%)	0 (0%)	2 (1.5%)
Relationship status, *N* (%)			
Single	83 (25.1%)	55 (27.5%)	28 (21.0%)
Living with spouse/partner	196 (59.2%)	110 (55.0%)	86 (65.6%)
Divorced/widowed/living separately from spouse	52 (15.7%)	35 (17.0%)	17 (13.0%)
# Adults living in home, mean (sd)	1.1 (1.3)	1.1 (1.5)	1.2(0.9)
# Children living in home, mean (sd)	0.4 (0.9)	0.4 (0.8)	0.5 (0.9)
Habits on most days, mean (sd)			
½ plate of vegetables	2.9 (1.0)	2.8 (1.0)*	3.1 (1.0)*
Brisk walk	2.9 (1.0)	2.8 (1.0)	3.0 (0.9)
Pause before reacting	3.3 (0.8)	3.4 (0.7)	3.3 (0.9)
Notice sensory experiences	3.9 (0.9)	3.9 (0.9)	3.8 (0.8)
Social support for diet, mean (sd)			
Family encouragement	13.8 (6.0)	13.8 (6.1)	3.8 (5.8)
Friend encouragement	10.7 (4.9)	10.3 (4.6)*	11.4 (5.3)*
Social support for physical activity, mean (sd)			
Friend participation	13.1 (6.4)	13.1 (6.4)	13.1 (6.5)
Family participation	15.7 (6.9)	15.8 (6.9)	15.7 (7.0)
Family rewards	3.8 (1.4)	3.7 (1.2)	4.0 (1.7)
Quality of life, mean (sd)			
Energy/vitality	58.0 (19.7)	59.3 (18.4)	55.9 (19.1)
Mental health	80.1 (12.3)	80.9 (12.0)	78.9 (12.7)
Perceived stress, mean (sd)	19.7 (6.7)	19.18 (6.8)	20.5 (6.5)
Date of baseline survey, *N* (%)			
Before COVID (March 11, 2020)	125 (37.8%)	80 (40.0%)	45 (34.4%)
During 1st peak COVID (March 11, 2020–May 1, 2021)	143 (43.2%)	84 (43.0%)	59 (45.0%)
After 1st peak COVID (May 1, 2021)	63 (19.0%)	36 (18.0%)	27 (20.6%)
Matched to preferred group, *N* (%)	N/A	93 (46.5%)	N/A

*p ≤ 0.05

Table 2 presents the multivariable models. The base model including only the sociodemographic covariates shows that only fewer years of education remained a correlate of no preference (adjusted OR, 0.92; 95% CI, 0.84–0.999; *p* = 0.047). Model 1 adds the strength of the habit of eating vegetables on most days and shows that it is associated with having no preference independently of education (adjusted OR, 1.29; 95% CI, 1.02–1.63;* p* = 0.03). Model 2 eliminates the strength of the habit of eating vegetables, adds encouragement from friends for a healthy diet, and shows that it is not significantly associated to preference (adjusted OR, 1.05; 95% CI, 1.00–1.11; *p* = 0.051). Model 3 includes both the strength of the habit of eating vegetables and encouragement from friends for a healthy diet and shows that the habit of eating vegetables remains significant (OR, 1.27; 95% CI, 1.01–161; *p* = 0.04).

**Table 2:** Multivariable predictors of no preference^a^

**Variables**	**Base model**^**b**^		**Model 1**^**c**^		**Model 2**^**d**^		**Model 3**^**e**^	
	**OR**	**95% CI**	**OR**	**95% CI**	**OR**	**95% CI**	**OR**	**95% CI**
Female	1.39	0.82–2.37	1.40	0.82–2.90	1.24	0.72–2.13	1.25	0.72–2.16
Age, years	0.99	0.97–1.01	0.99	0.96–1.01	0.99	0.97–1.01	0.99	0.97–1.01
Hard to pay for basics (ref: not hard)	1.02	0.52–2.00	1.03	0.52–2.02	0.99	0.50–1.95	1.00	0.50–1.97
Education, years	0.92*	0.84–0.999*	0.92	0.84–1.00	0.92	0.85–1.01	0.92	0.85–1.01
Caucasian non-Hispanic (ref: else)	1.03	0.62–1.73	1.06	0.63–1.77	1.16	0.68–1.98	1.18	0.69–2.01
Single (ref: divorced/widowed/living separately)	0.98	0.45–2.13	1.00	0.46–2.19	1.00	0.46–2.18	1.00	0.46–2.22
Living with spouse/partner (ref: divorced/widowed/living separately)	1.70	0.86–3.38	1.75	0.88–3.48	1.74	0.87–3.47	1.77	0.89–3.56
*Habit: ½ plate of vegetables*	–-	–-	1.29*	1.02–1.63*	–-	–-	1.27*	1.01–1.61*
Friend encouragement for diet	–-	–-	–-	–-	1.05	1.00–1.11	1.05	0.996–1.10

*p ≤ 0.05

^a^Analytic sample included 325 participants because 6 participants refused to provide information on gender, race/ethnicity, and difficulty paying for basics

^b^Adjusted for gender, age, ability to pay for basics, education, race/ethnicity, and relationship status

^c^Adjusted for base model variables and habit of eating ½ plate of vegetables

^d^Adjusted for base model variables and friend encouragement for diet

^e^Adjusted for base model variables, habit of eating ½ plate of vegetables, and friend encouragement for diet

### Preference for Group-Based vs. Self-directed

Table 3 is limited to the subgroup of participants with a preference (*n* = 200) and presents unadjusted comparisons between a preference for group-based (*n* = 144) or self-directed treatment (*n* = 56). Those who preferred self-directed treatment had fewer years of education (15.5 vs. 16.5 years, *p* = 0.03), a stronger habit of eating vegetables on most days (3.0 vs. 2.7,* p* = 0.04), a stronger habit of taking a brisk ≥ 30-min walk on most days (3.1 vs. 2.7, *p* = 0.04), and greater encouragement for a healthy diet from family (15.2 vs. 13.3, *p* = 0.05).

**Table 3:** Baseline comparison of participants with preference for group-based vs. self-directed treatment

	**Prefer group-based** ***N* = 144**	**Prefer self-directed** ***N* = 56**
Age (years), mean (sd)	56.2 (11.1)	55.2 (11.5)
Female, *N* (%)	107 (74.3%)	35 (64.8%)
Education (years), mean (sd)	16.5 (2.7)*	15.5 (2.9)*
Race/ethnicity, *N* (%)		
Hispanic	10 (6.9%)	5 (9.3%)
Black or African American, non-Hispanic	34 (23.6%)	10 (18.5%)
Caucasian, non-Hispanic	90 (62.5%)	36 (66.7%)
Other, non-Hispanic	10 (6.9%)	3 (5.6%)
Refused	1 (0.7%)	1 (1.8%)
Ability to pay for basics, *N* (%)		
Very hard/somewhat hard	20 (13.9%)	9 (16.1%)
Not hard at all	124 (86.1%)	47 (83.9%)
Relationship status, *N* (%)		
Single	43 (29.9%)	12 (21.4%)
Living with spouse/partner	74 (51.4%)	36 (64.3%)
Divorced/widowed/ living separately from spouse	27 (18.8%)	8 (14.3%)
# Adults living in home, mean (sd)	1.1 (1.7)	1.1 (0.9)
# Children living in home, mean (sd)	0.4 (0.8)	0.4 (0.9)
Habits on most days, mean (sd)		
½ plate of vegetables	2.7 (1.0)*	3.0 (0.9)*
Brisk walk	2.7 (1.0)*	3.1(1.0)*
Pause before reacting	3.4 (0.7)	3.4 (0.8)
Notice sensory experiences	3.8 (1.0)	3.9 (0.8)
Social support for diet, mean (sd)		
Family encouragement	13.3 (5.9)*	15.2 (6.3)*
Friend encouragement	10.5 (4.7)	9.6 (4.3)
Social support for physical activity, mean (sd)		
Friend participation	13.3 (6.6)	12.5 (5.9)
Family participation	15.3 (7.0)	17.0 (6.5)
Family rewards	3.6 (1.1)	3.9 (1.5)
Quality of life, mean (sd)		
Energy/vitality	59.2 (19.0)	59.7 (17.1)
Mental health	80.6 (12.6)	81.9 (10.4)
Perceived stress, mean (sd)	19.2 (7.1)	19.0 (6.2)
Date of baseline survey, *N* (%)		
Before COVID (March 11, 2020)	59 (41.0%)	21 (37.5%)
During 1st peak COVID (March 11, 2020–May 1, 2021)	59 (41.0%)	25 (44.6%)
After 1st peak COVID (May 1, 2021)	26 (18.1%)	10 (17.9%)
Matched to preferred group, *N* (%)	69 (47.9%)	24 (42.9%)

*p ≤ 0.05

Table 4 presents the multivariable models. The base model including only the sociodemographic variables shows that fewer years of education remained associated with preference for self-directed treatment (adjusted OR, 0.88; 95% CI, 0.78–0.998; *p* = 0.046). Model 1 adds the strength of the habit of eating vegetables on most days and shows that it is associated with preference for self-directed treatment independently of education (adjusted OR, 1.60; 95% CI, 1.13–2.26; *p* = 0.01). Model 2 eliminates the strength of the habit of eating vegetables and adds the strength of the habit of taking a brisk walk and shows there is no significant association with preference for self-directed treatment (adjusted OR, 1.37; 95% CI, 0.97–1.91; *p* = 0.07) and eliminated the association of education. Model 3 eliminated both the habit of eating vegetables and the habit of taking a brisk walk and showed that encouragement from family for a healthy diet was not associated with preference for self-directed treatment independent of education (adjusted OR, 1.05; 95% CI, 0.99–1.11;* p* = 0.12). Model 4 included all sociodemographic factors and potential correlates and showed that only the strength of the habit of eating vegetables remained a significant correlate of a preference for self-directed treatment independent of education (adjusted OR = 1.55; 95% CI, 1.09–2.22; *p* = 0.02).

**Table 4:** Multivariable predictors of preference for self-directed treatment^a^

**Variables**	**Base model**^**b**^		**Model 1**^**c**^		**Model 2**^**d**^		**Model 3**^**e**^		**Model 4**^**f**^	
	**OR**	**95% CI**	**OR**	**95% CI**	**OR**	**95% CI**	**OR**	**95% CI**	**OR**	**95% CI**
Female	0.71	0.34–1.46	0.71	0.34–1.59	0.75	0.36–1.56	0.72	0.34–1.48	0.74	0.35–1.58
Age, years	1.00	0.97–1.03	1.00	0.97–1.03	1.00	0.97–1.03	1.00	0.97–1.03	1.00	0.97–1.03
Hard to pay for basics (ref: not hard)	1.12	0.43–2.88	1.25	0.47–3.36	1.18	0.45–3.11	1.13	0.43–2.97	1.33	0.49–3.61
Education, years	0.88*	0.78–0.998*	0.87*	0.77–0.99*	0.90	0.79–1.02	0.89	0.78–1.00	0.89	0.78–1.00
Caucasian, non-Hispanic (ref: else)	1.19	0.57–2.46	1.28	0.61–2.70	1.20	0.57–2.51	1.33	0.63–2.83	1.43	0.66–3.10
Single (ref: divorced/widowed/living separately)	1.06	0.36–3.11	1.10	0.37–3.32	1.04	0.55–3.10	1.14	0.38–3.37	1.15	0.38–3.53
Living with spouse/partner (ref: divorced/widowed/living separately)	1.46	0.57–3.72	1.54	0.59–4.00	1.41	0.55–3.62	1.35	0.52–3.48	1.39	0.52–3.69
Habit: ½ plate of vegetables	–-	–-	1.60*	1.13–2.26*	–-	–-	–-	–-	1.55*	1.09–2.22*
Habit: brisk walk	–-	–-	–-	–-	1.37	0.97–1.91	–-	–-	1.26	0.88–1.78
Family encouragement for diet	–-	–-	–-	–-	–-	–-	1.05	0.99–1.11	1.04	0.98–1.10

*p ≤ 0.05

^a^Analytic sample included 200 participants because 3 participants refused to provide information on gender and race/ethnicity

^b^Adjusted for gender, age, ability to pay for basics, education, race/ethnicity, and relationship status

^c^Adjusted for base model variables and habit of eating ½ plate of vegetables

^d^Adjusted for base model variables and habit of taking a brisk walk

^e^Adjusted for base model variables and family encouragement for diet

^f^Adjusted for base model variables, habit of eating ½ plate of vegetables, habit of taking a brisk walk, and family encouragement for diet

## Discussion

Behavioral randomized clinical trials are rarely double-blind because participants generally know what treatment they are in. Thus, for behavioral trials it is important to understand more about patient preference for a particular treatment and how it may affect the quality of the trial. The purpose of the current study was to determine relative frequency and correlates of patient preference for an in-person group-based or a remote self-directed, lifestyle intervention prior to random assignment to one of these treatment groups. The major finding was that pre-existing healthy lifestyle habits were associated with having no preference and, in those with a preference, a preference for self-directed treatment.

Forty percent of the participants in this cohort had no preference for a particular treatment. This is consistent with a meta-analysis that found 44% of participants had no preference [2]. However, in contrast to past findings we did not find a gender effect on preference, and having no preference was associated with lower rather than higher education [5]. It is possible that those with less education had higher levels of trust that either treatment option would be equally effective, particularly in light of the information session they attended before enrollment that provided details about both arms and emphasized the equipoise of not knowing which arm was more effective. Alternatively, since this cohort is highly educated with an average of 15.9 years of education, any association may have been attenuated by limitations in diversity on education. Although we expected to see an association between the evolution of the COVID pandemic and a preference for a remote self-directed treatment that did not require in-person contact, we failed to find any association. A preference for self-directed treatment was stable across the pandemic.

Most striking about these findings was that the strength of a pre-existing health habit of daily vegetable intake was associated with both having no preference for a particular treatment and, if there was a preference, having a preference for self-directed treatment. Individuals with a healthier lifestyle may believe that they can be successful without the additional support that a group offers because they already have healthy habits. While the unadjusted comparison showed a difference in preference based on friend support for diet, it did not retain significance when habits were considered in the same model. This is consistent with the hypothesis that individual habits are a more important factor in preference than the support for those habits.

Precision lifestyle medicine argues for a close alignment between the needs of the patient and the offerings of the treatment [15]. These findings provide insight on how to accomplish this alignment. Individuals who are already engaged in the process of optimizing their health may have less interest in, and need for, structured help from professionals. Conversely, those with weaker health habits may be more likely to prefer structured, in-person group support. This would imply that a stepped care approach to lifestyle interventions is optimum where those with more behavioral resources need less intensive treatment and those with fewer of these resources may need more intensive treatment. This could save healthcare resources if more costly help from professionals was directed to those at an earlier step in the process of lifestyle change.

There are several limitations to this study. The study population is not representative of all people diagnosed with metabolic syndrome. The study sample had a high educational attainment as well as a majority having no difficulty paying for basic. Higher education and socioeconomic status could have impacted some of the other baseline characteristics that assess stress, habits, and support. Other limitations were the tools used to measure healthy habits. While the Self-Report Habit Index is a validated tool, there may be other aspects of healthy habit that were not captured in this tool. Finally, this study sample size consisted of half of the full ELM cohort due to ongoing recruitment at the time of analysis. The full cohort will be used for future analysis of how patient preferences impact clinical outcomes.

The next step in this research is to investigate the association between patient preference and clinical outcome. There are two additional questions that arise: (1) Can the very fact of having a preference for one of the treatments influence outcomes? (2) Can getting the preferred treatment (or being denied it) influence outcomes [4]? Having a preference for a particular treatment has been associated with better clinical outcome in some [2, 3] but not all [16, 17] studies. Although concordance between preference for a particular treatment and receipt of it has been hypothesized to be beneficial on outcomes [3, 18], there is little empirical evidence for benefit either on outcomes or internal validity [2, 18, 19]. One study even found that discordance between preferred and received treatment produced better outcomes than concordance [20]. Thus, while it is possible that having a preference benefits clinical outcomes, it is unlikely that concordance between preferred and received treatment also provides benefit. To answer these questions, a follow-up to the present report is planned where we will assess the impact of preference on such outcomes as remission of the MetS, weight loss, and improvement in such health behaviors as diet, physical activity, and mindful attention to eating and physical activity.

In summary, the pre-existing habit of eating vegetables was associated with no preference and preference for a less intensive lifestyle treatment. Post-treatment follow-up of the trial results will determine if concordance between preference and treatment assignment influences outcomes.

## Data Availability

Data and materials used in this study are available from the corresponding author upon reasonable request.

## Code Availability

Code used in this study is available from the corresponding author upon reasonable request.

## Declarations

### Disclaimer

The Fund had no role in the design or conduct of the trial, the decision to publish trial results, the analysis of data, or the preparation of this manuscript.
